# Functional Characterization of the Canine Heme-Regulated eIF2*α* Kinase: Regulation of Protein Synthesis

**DOI:** 10.1155/2009/251915

**Published:** 2009-06-22

**Authors:** Kimon C. Kanelakis, Jayashree Pyati, Pamela C. Wagaman, Jui Chang Chuang, Young Yang, Nigel P. Shankley

**Affiliations:** ^1^Department of Internal Medicine, Johnson & Johnson Pharmaceutical Research & Development L.L.C., Merryfield Row 3210, San Diego, CA 92121, USA; ^2^Shire HGT, 700 Main St., Cambridge, MA 02139, USA

## Abstract

The heme-regulated inhibitor (HRI) negatively regulates protein synthesis by phosphorylating eukaryotic initiation factor-2*α* (eIF2*α*) thereby inhibiting protein translation. The importance of HRI in regulating hemoglobin synthesis in erythroid cells makes it an attractive molecular target in need of further characterization. In this work, we have cloned and expressed the canine form of the HRI kinase. The canine nucleotide sequence has 86%, 82%, and 81% identity to the human, mouse, and rat HRI, respectively. It was noted that an isoleucine residue in the ATP binding site of human, rat, and mouse HRI is replaced by a valine in the canine kinase. The expression of canine HRI protein by in vitro translation using wheat germ lysate or in Sf9 cells using a baculovirus expression system was increased by the addition of hemin. Following purification, the canine protein was found to be 72 kD and showed kinase activity determined by its ability to phosphorylate a synthetic peptide substrate. Quercetin, a kinase inhibitor known to inhibit mouse and human HRI, inhibits canine HRI in a concentration-dependent manner. Additionally, quercetin is able to increase de novo protein synthesis in canine reticulocytes. We conclude that the canine is a suitable model species for studying the role of HRI in erythropoiesis.

## 1. Introduction

Heme-regulated inhibitor (HRI) is an eIF2*α* kinase, which belongs to the eIF2*α* kinase subfamily [1] that includes the double-stranded RNA-dependent eIF2*α* kinase (PKR), the general control of nitrogen metabolism kinase (GCN2), and them endoplasmic reticulum resident kinase, PKR-related kinase (PERK), which is identical to the enzyme pancreatic eIF2*α* kinase (PEK) that is highly expressed in pancreas [2]. Sequence comparison between HRI and other eIF2*α* kinases reveals that the eIF2*α* kinases share some degree of homology in their kinase catalytic domains. However, between the N- and C-lobes HRI has a distinct kinase insert that contains a heme binding site [3]. Kinase activity of HRI is modulated by binding of heme to the heme-binding domains located at its kinase insert and the amino terminus. As an eIF2*α* kinase, HRI specifically phosphorylates eIF2*α* at residue Serine 51 [4], blocking GTP exchange required for the recycling of eIF2, and thus inhibits protein synthesis [5]. Because HRI is predominantly expressed in erythroid cells, HRI exerts its kinase activity to regulate the synthesis of hemoglobin [6]. 

Phenotypic changes in HRI knockout mice show that HRI is nonessential, but plays an important regulatory role by dictating hemoglobin synthesis in erythroid cells [1]. Notably, in the absence of HRI kinase activity, reticulocytes obtained from *HRI*-deficient mice had a higher rate of protein synthesis. The importance of HRI in the pathophysiology of heme-deficiency disorders such as anemia of iron deficiency, protoporphyria, and *β*-thalassemia has been recently shown in animal models [7]. 

Interestingly, HRI does not exist in yeast, *Drosophila melanogaster*, and *C. elegans* [5, 8]. It is postulated that HRI has evolved during the development of the circulation to meet the increasing demand for oxygen in larger organisms. Alignment of the amino acids of HRI from human, mouse, rat, and rabbit [8] reveals that HRI is conserved throughout evolution among these mammals with approximately 77% identity and 83% homology. Because of the importance of HRI in regulating hemoglobin synthesis in mammals, we sought to evaluate the feasibility of using the canine as a model for studying the homeostasis of erythropoiesis. The canine HRI was cloned, expressed by in vitro transcription and translation in wheat germ lysate and also in baculovirus protein expression system and then compared to the murine and human HRI proteins by its functional activity and inhibition by a kinase inhibitor. Additionally, the function of HRI on protein synthesis was evaluated in canine blood with the kinase inhibitor, quercetin.

## 2. Material and Methods

### 2.1. Tissue Isolation and Total RNA Preparation

All procedures were performed according to the internationally accepted guidelines for the care and use of laboratory animals in research and were approved by the local International Animal Care and Use Committee. Canine spleen was collected and RNA stabilization was achieved by submersion of tissue in RNA*later* (Ambion, Milan, Italy). Total cellular RNA (tc-RNA) from spleen was extracted using the RNeasy kit following manufacturer's instructions (Qiagen, Chatsworth, Calif, USA).

### 2.2. Cloning of Canine HRI cDNA

Reverse transcription (RT) reactions on human (Clontech, Mountain View, Calif, USA) and canine tc-RNA were performed in a 20 *μ*L reaction mixture containing 10 mM Tris-HCl (pH 8.4), 50 mM KCl, 5 mM MgCl_2_, 500 *μ*M dNTP, 1.25 *μ*M of oligo(dT) primer, 5 *μ*g tc-RNA, 40 U of RNase inhibitor, 2 U of RNaseH, and 50 U of reverse transcriptase II (Invitrogen, Carlsbad, Calif, USA). The RT reactions were performed under the following conditions: 90 minutes at 42°C, 10 minutes at 70°C, followed by 20 minutes at 37°C. The resulting cDNA samples (1 *μ*L) were used for PCR amplification with the addition of 45 *μ*L of Supermix (Invitrogen, Carlsbad, Calif, USA) containing 2.2 U of Taq DNA polymerase (a mixture of recombinant Taq DNA polymerase and DNA polymerase from pyrococcus species GB-D) in 66 mM Tris-SO_4_ (pH 9.1 at 25°C), 19.8 mM (NH_4_)_2_SO_4_, 2.2 mM MgSO_4_, 220 *μ*M dGTP, 220 *μ*M dATP, 220 *μ*M dTTP, 220 *μ*M dCTP, with stabilizers and 20 *μ*M of sense [5′-AACGTTGAATTCGCCACCATGCTGGGGGGCAGCTCCGGT-3′] and antisense primers [5′-AACGTTGCGGCCGCTTAAGGCAGGACGGGCACAGTC-3′]. Oligonucleotide primers were designed to be homologous to the corresponding region of the human HRI (GenBank Accession Number: NM_014413). The cDNA fragments were amplified by PCR under the following conditions: 30 s at 94°C for 1 cycle, followed by 94°C for 30 seconds, 30 seconds at 60°C, 72°C for 3 minutes for 30 cycles.

### 2.3. Cloning of Canine HRI cDNA into Expression Vectors

Two chimeric oligonucleotide primers (described earlier) were synthesized to facilitate the sub-cloning step (Allele Biotech, San Diego, Calif, USA). The chimeric 5′ primer included three adjacent upstream nucleotides; 6 random bp followed by a 6 bp EcoRI restriction enzyme sequence and a 6 bp Kozak sequence leading to the 21 nucleotides complementary to the canine HRI cDNA sequence. The chimeric 3′ primer included 6 random bp followed by a 6 bp NotI restriction enzyme sequence and 22 bp complementary to the canine HRI cDNA sequence. PCR with the above 2 chimeric primers resulted in a 1925 bp product, consisting of full length HRI and the sequences corresponding to the chimeric primers.

### 2.4. Sequencing

Recombinant double-stranded plasmids containing the 1893 bp full-length HRI cDNA insert served as templates for cycle sequencing with specific forward and reverse primers and fluorescence-based dideoxynucleotides, using the dideoxy-terminator cycle sequencing kit (Perkin Elmer, Applied Biosystems, Foster City, Calif, USA). HRI cDNA sequences were determined by the use of a DNA Sequencer (ABI Model 373, Applied Biosystems). Canine HRI cDNA sequences were validated by sequencing cDNA products from three separate RT-PCR reactions, primers were for the forward reaction: 5′-ATCTGATGTCCCAGCAGAACTCCA-3′, 5′-GATACGGAAGAGTGTACAAGGTCA-3′, 5′-AGGATCCGACTATGACGCC-3′, and 5′-TGCACATCCAGATGCAGCTGTGT-3′, and for the reverse:, 5′- GAGCTCTAGCAGGATCACACCC-3′, 5′-GATCTTGTGCATGGGTCACGTGAA-3′, 5′-GCTGGGACATCAGATTCATCATACT-3′, (all primers purchased from Allele Biotech).

### 2.5. Preparation of Recombinant HRI Baculovirus and Expression of Canine HRI Protein in Sf9 Cells

Expression of canine HRI protein (cHRI) in Sf9 cells was achieved using the protocol from a commercially available Bac-n-Bac transfection kit (Invitrogen). The pFastBac transfer vector, in which the canine HRI cDNA was inserted, was transformed into DH10Bac competent cells containing bacmid DNA and a helper plasmid. Cells were incubated on ice for 30 minutes followed by 60 seconds at 42°C allowed for transposition of the pFastBac-cHRI plasmid with the bacmid catalyzed by the transposition sequences on the helper plasmid. Colonies containing recombinant bacmids were identified by selection of blue/white colonies caused by disruption of the lacZ*α* gene following the cHRI cDNA insertion. 

The bacmid DNA was isolated and then transfected into Sf9 cells to make recombinant baculovirus as previously described [9]. Sf9 cells were grown in Sf-900 II serum-free medium in suspension cultures with continuous shaking (150 rpm). Cultures were infected in log phase of growth with recombinant baculovirus at the multiplicity of infection of 3.0. Cells were harvested after 48 hours of infection, washed in phosphate buffered saline solution, resuspended in 1 volume of buffer (10 mM Hepes, pH 7.5, 300 mM NaCl, 5 mM MgCl_2_, 10% glycerol) containing protease inhibitor mixture, and ruptured by sonication. The lysate was then centrifuged at 17,000 ×g for 20 minutes at 4°C and the supernatant collected.

### 2.6. Purification of Canine HRI Protein

Canine, mouse, rat and human HRI proteins were expressed in Sf9 cells and purified by Ni-NTA affinity chromatography (Qiagen, Valencia, Calif, USA) according to the manufacturer's recommendations and dialyzed in buffer (5 mM Hepes, pH 7.4, 100 mM NaCl, 2 mM DTT) overnight. The canine HRI protein was separated by SDS-PAGE and identified by Western blotting using a mouse anti-histidine primary antibody at 1:1000 (Clontech, Mountain View, Calif, USA) and a secondary anti-mouse HRP conjugated antibody at 1:10,000 (Pierce Biotechnology, Rockford, Ill, USA) followed by enhanced chemiluminescence using the Amersham Enhanced Chemiluminescence Plus Western blotting detection system (GE Biosciences, Piscataway, NJ, USA).

### 2.7. Expression of Canine HRI Protein by TNT

The cloned canine HRI in the pcDNA4 vector was linearized with NotI and 2.8 *μ*g of the linearized DNA was used for transcription/translation (TNT). Protein expression of the canine HRI was achieved using the cell-free expression kit from Promega using wheat germ lysate according to the manufacturer's protocol. The reaction mix was supplemented with 0.5 mM hemin to increase expression of functional kinase. The [^35^S]-canine HRI was immunoprecipitated with an anti-histidine antibody bound to protein A-sepharose and then separated on a 4–20% PAGE and visualized by autoradiography.

### 2.8. Kinase Activity Determination of Purified Canine HRI Protein

The purified canine HRI protein was used to determine its kinase activity in a nonradioactive, phosphorylation-dependent, homogeneous assay, AlphaScreen [10]. Briefly, biotinylated HRI substrate, Poly-GT (Cisbio International, Bedford, Mass, USA) was incubated with purified cHRI kinase for 2 hours at room temperature, in the presence of 4 *μ*M ATP, and 5 mM Mg^2+^/Mn^2+^ allowing for substrate phosphorylation by cHRI. AlphaScreen donor and acceptor beads were then added to the reaction mix according to the manufacturer's protocol (Perkin-Elmer, Waltham, Mass, USA). Kinase activity was determined by detecting an output signal at 540 nm wavelength, with signal intensity being directly proportional to phosphorylated substrate. 

For inhibition studies, quercetin (Sigma Chemicals, St. Louis, Mo, USA) was incubated with the kinase in the kinase activity assay as detailed earlier. Inhibition of phosphorylation by the inhibitor was measured as a decrease in AlphaScreen signal. The pIC_50_ values were determined by fitting a three-parameter logistic function using GraphPad Prism, version 4.02 (Graph Pad Software, San Diego, Calif, USA). Data are presented as mean ± SEM of triplicate values.

### 2.9. Protein Synthesis in Peripheral Canine Blood

Whole blood from beagle obtained from Bioreclamation (Hicksville, NY, USA) was washed twice with ice-cold PBS containing 5 mM glucose by centrifuging at 2000 rpm for 5 minutes at 4°C to remove platelets and clotting factors and was passed through a cell strainer (70 *μ*M pore size, BD Biosciences, Lexington, Ky, USA) to remove any clots. The blood cell pellet was then resuspended in pre-warmed (37°C) methionine-free DMEM (Hyclone, Logan, Utah, USA) containing 2 mM glutamine and was treated with either vehicle (1% DMSO) or quercetin in the presence of 0.2 mCi [^35^S]-methionine (Amersham Bioscience Inc., Piscataway, NJ, USA) for 4 hours at 37°C in 50 mL centrifuge tubes. The samples were swirled every 30 minutes to ensure adequate mixing. After labeling, the cells were transferred to Eppendorf tubes and washed with ice-cold PBS by centrifugation at 14,000 rpm for 5 minutes at 4°C. The cell pellets were lysed in 1 mL of PBS containing 1% Triton X-100. To remove free [^35^S]-methionine, the lysates were subjected to TCA precipitation and centrifuged at 14,000 rpm for 10 minutes at 4°C. Methionine incorporation into protein was determined by addition of Scintiverse liquid scintillation cocktail and then the [^35^S] signal was measured using a beta-counter (Beckman Coulter, Fullerton, Calif, USA).

## 3. Results

### 3.1. Cloning and Sequence Comparison of Canine HRI cDNA with other mammalian HRI

**Figure 1:**
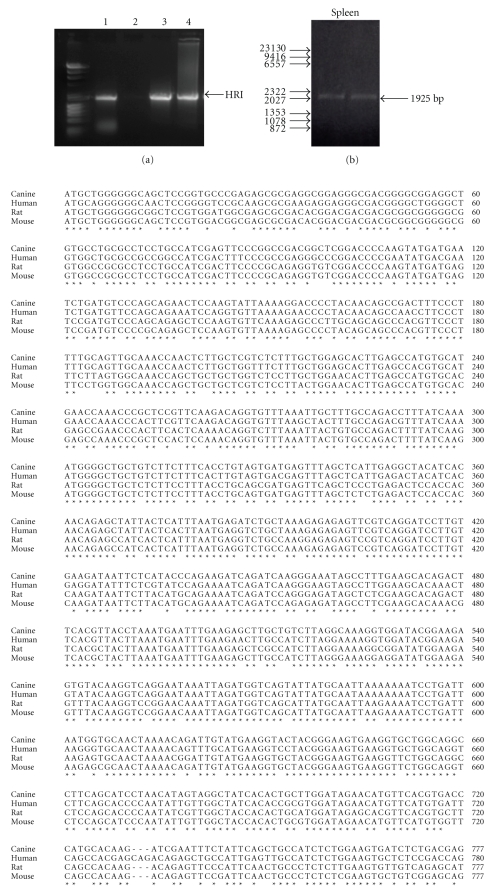

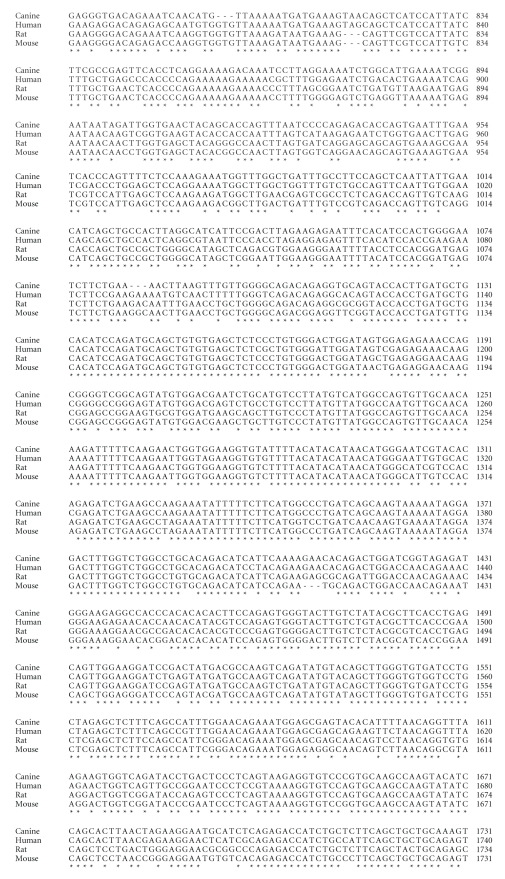

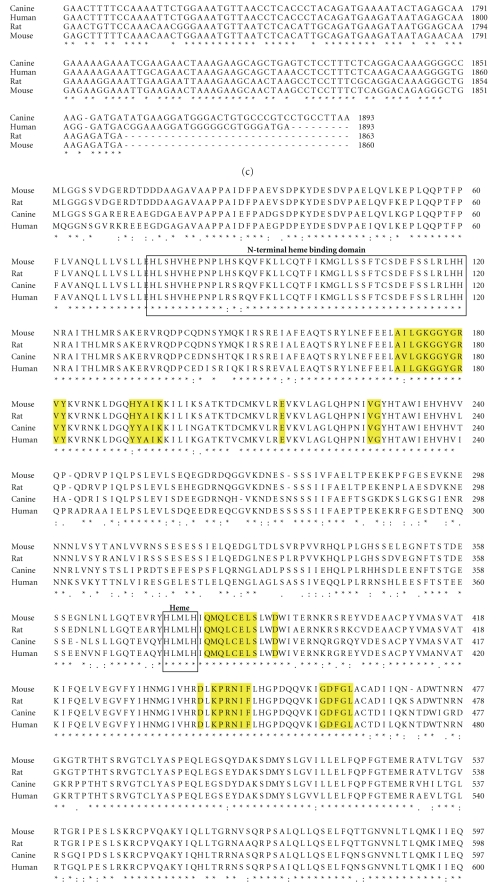

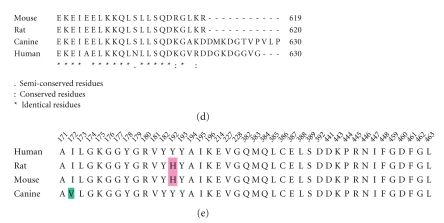
(a) Expression pattern of HRI in human tissues. RT-PCR products from human tissue using HRI specific primers were separated on a 0.8% agarose gel and visualized by UV. Lanes: 1, liver; 2, kidney; 3, lymph node; 4, spleen. (b) RT-PCR products of canine HRI cDNA from spleen. 50 ng of the resulting cDNA PCR products were separated on an agarose gel (0.8%), stained with ethidium bromide and visualized by UV. (c) Nucleotide sequence comparison of the canine HRI with human, rat and mouse HRI. Conserved nucleotides between all four species are denoted with a star. (d) Amino acid sequence alignment of the canine HRI with the mouse, rat, and human HRI. Semi-conserved residues are designated with a period underneath, conserved residues are shown by semicolon underneath, and the identical residues have stars underneath them. The residues important for ATP binding are highlighted in gray. (e) Alignment of the residues involved in ATP binding. Residues important for ATP binding for the human, mouse, rat, and canine HRI are shown. Divergences between the species are highlighted.

To decide which canine tissues to use for cloning canine HRI cDNA, we performed RT-PCR for HRI using human liver, kidney, lymph node or spleen (Clontech, Mountain View, Calif, USA). As shown in Figure 1(a), HRI was detected in liver, lymph node, and spleen, but not detected in the kidney. Because previous reports demonstrated that HRI is predominantly expressed in erythroid-derived cells [6, 7], we presume that the presence of peripheral blood in the tissues allowed for the detection of HRI. Based on the tissue expression results of the human HRI we decided to clone the canine HRI from canine spleen.

To clone canine HRI cDNA, we prepared total RNA from canine spleen followed by first strand cDNA synthesis using oligo(dT) primers (Invitrogen). The resulting cDNA was used in RT-PCR with cloning primers designed based on the canine genomic sequence corresponding to the human HRI sequence. A PCR fragment of the expected 1925 bp was amplified (Figure 1(b)). The resulting fragment was sequenced and confirmed to be the canine HRI (GenBank Accession Number FJ911905), showing 86% identity in nucleotide sequence to the human, 82% identity to the mouse and 81% identity to the rat HRI (Figure 1(c)). At the amino acid level the canine protein shows 83% identity to the human HRI sequence (NM_014413) and 81% to the mouse (NM_013557) and rat HRI (NM_013223) (Figure 1(d)). As shown in Figure 1(e), the canine sequence shows only 1 amino acid difference in the ATP-binding site compared to the human sequence. The high conservation in the residues involved in ATP binding between the human, canine and rodent proteins is consistent with the functional importance of HRI and its conservation throughout evolution.

### 3.2. Expression of Canine HRI Protein by TNT

**Figure 2:**
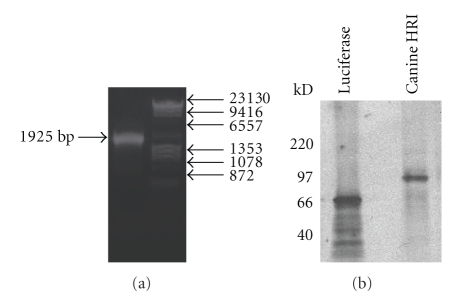
(a) Restriction digestion analysis of the canine-HRI-containing pcDNA4 plasmid DNA verifies the canine HRI was inserted into the NotI and EcoRI restriction sites. Digested fragments were separated by agarose gel electrophoresis (0.8%) and found to have a size of 1925 bp. (b) Canine HRI expressed by TNT using a Promega Wheat Germ Lysate kit. Lane 1 shows the [^35^S]-luciferase control and Lane 2 shows the expression of [^35^S]-canine HRI. The molecular weight markers are shown to the left of the autoradiography.

To express canine HRI protein, the canine HRI cDNA was cloned into the pcDNA4 vector for expression by TNT (Figure 2(a)). After the pcDNA4-HRI was restriction enzyme digested with NotI, the linearized DNA was incubated with wheat germ lysate and [^35^S]-methionine to produce the radiolabeled canine HRI protein by TNT. The resulting translated products were analyzed on a 4–20% Tris-glycine gel followed by autoradiography. We found that the canine HRI protein yield was very low using standard TNT reaction conditions (data not shown). When 0.5 mM hemin was included in the TNT reaction mix, the yield of the canine protein was increased by ~5-fold (Figure 2(b)). The enhanced production of HRI by heme strongly suggests a functional form of canine HRI is expressed from the cloned canine HRI cDNA.

### 3.3. Expression of Canine HRI Proteins by Baculovirus Expression System

**Figure 3:**
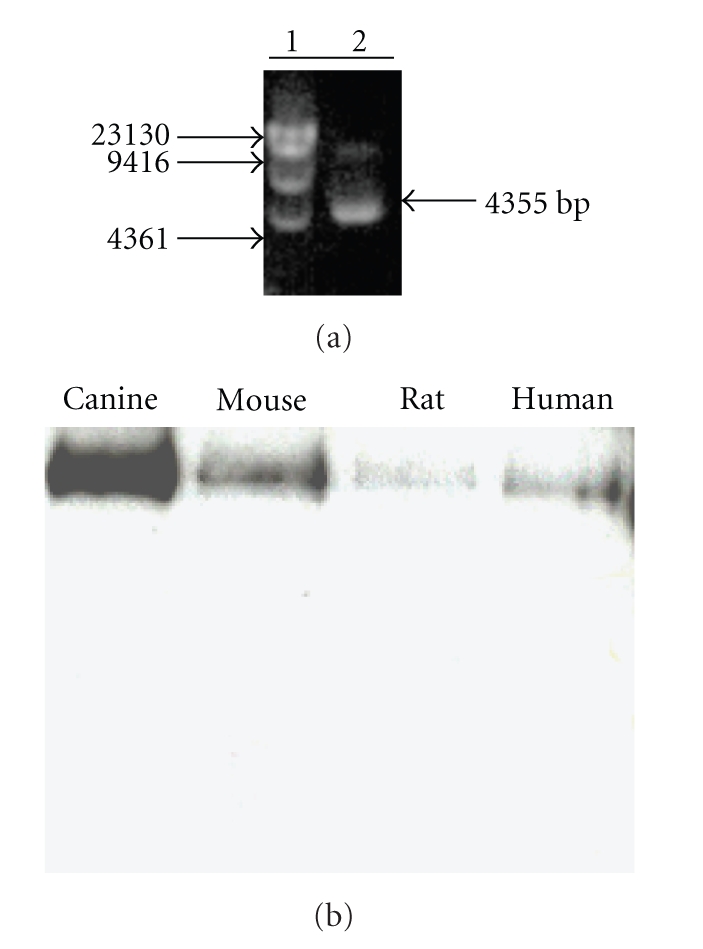
(a) Restriction digestion analysis of the canine HRI-bacmid DNA verifies the insertion of canine HRI cDNA into the bacmid DNA with correct orientation. Lane 1, Molecular Weight markers; Lane 2, pFastBac-canine HRI cDNA. (b) Expression of mammalian HRIs. HRI, expressed in insect cells, was detected by Western blot analysis using an affinity purified mouse monoclonal antibody against histidine and visualized by enhanced chemiluminescence. Lane 1, canine HRI; Lane 2, mouse HRI; Lane 3, rat HRI; Lane 4, human HRI.

Since the majority of canine HRI protein produced by TNT was found to be misfolded and/or aggregated and exhibited very low kinase activity, we decided to express the canine HRI using an insect expression system. The canine HRI cDNA was inserted into the pFastBac vector and transformed into DH10Bac cells. After confirming that the canine HRI bacmid DNA contains the canine HRI cDNA insert by restriction digestion analysis, (Figure 3(a)), the bacmid DNA was transfected into Sf9 cells to produce recombinant canine HRI baculovirus. As shown in Figure 3(b), 48 hours postinfection of Sf9 cells with canine HRI baculovirus, a band of the expected molecular weight of 72 kD corresponding to the canine HRI, was isolated, following expression and purification by His6 affinity chromatography. Since this 72 kD protein bound to nickel affinity matrix and was specifically recognized by antibodies against either histidine (6xHis) (Figure 3(b)) or HRI (data not shown) epitopes, we concluded that this was the canine HRI. Similar to the expression of canine HRI protein by TNT, we found that canine HRI baculovirus infection resulted in low expression of the HRI protein if hemin was not included in the cell culture medium. When hemin at 0.5 mM was included, canine HRI protein expression in the baculovirus expression system was substantially increased. The human, mouse, rat, and canine proteins were expressed using the baculovirus expression system and used in comparison studies.

### 3.4. Kinase Activity of the Canine HRI Protein

**Figure 4:**
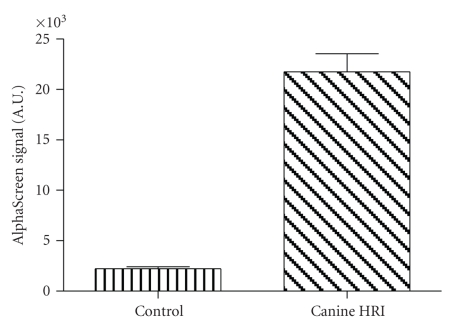
Kinase activity of recombinant canine HRI. Kinase activity of canine HRI was determined by an in-vitro kinase assay using purified canine HRI and a kinase peptide substrate. The experiment was performed in triplicate with the mean ± SEM shown.

**Figure 5:**
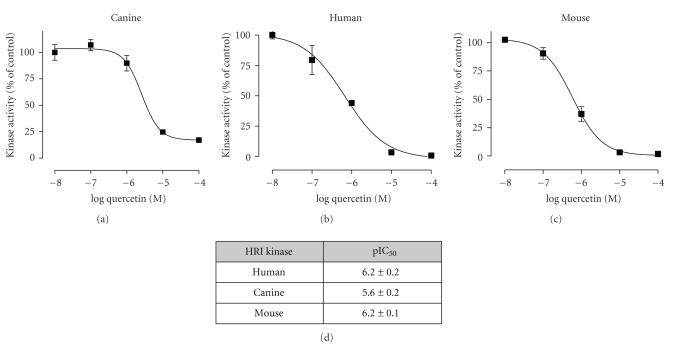
Inhibition of HRI kinase activity by quercetin. The kinase activity of the purified human, mouse and canine HRI proteins was determined using an in-vitro kinase assay in the presence of various concentrations of quercetin from 0.01 *μ*M to 100 *μ*M, shown in log M units. The data are expressed as a percent of activity compared to a vehicle control at 100% at the end of the two hour reaction. All experiments were performed in duplicate or triplicate with the mean ± SEM represented.

To determine if the purified canine HRI protein is enzymatically active, we performed a kinase activity assay by measuring the ability of the kinase to phosphorylate a peptide substrate. As shown in Figure 4, the canine HRI protein was able to effectively phosphorylate the peptide substrate. To investigate whether the high degree of homology in the ATP-binding site of canine, human, and rodent HRI results in similar binding of an ATP-competitive kinase inhibitor, we used a known human HRI kinase inhibitor, quercetin ([11] and our published data). Quercetin, reported to be an ATP-competitive kinase inhibitor [11], was tested at various concentrations from 0.01 *μ*M to 100 *μ*M against canine, human, and mouse HRI shown in Figure 5. We found that similar to human and mouse HRI, canine HRI kinase was inhibited with IC_50_ values equal to ~1 *μ*M by quercetin, demonstrating that among mammalian HRI, ATP-binding and catalysis are conserved.

### 3.5. Role of HRI in De Novo Protein Synthesis

**Figure 6:**
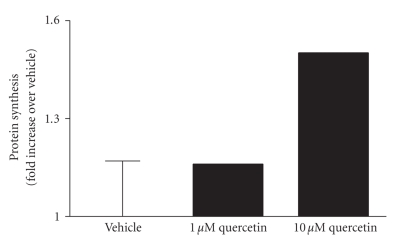
Effect of HRI inhibition on protein synthesis by quercetin in canine blood cells. Total protein synthesis in canine blood was determined by [^35^S]-methionine incorporation into newly synthesized protein in the presence of 1 or 10 *μ*M quercetin. The amounts of [^35^S]-methionine incorporation were compared to a vehicle control of 1% DMSO and the fold increase with the mean ± SEM from replicates is shown.

To determine the effect of HRI inhibition by quercetin on protein synthesis, we treated canine blood with either 1 or 10 *μ*M quercetin for 4 hours in the presence of [^35^S]-methionine (Figure 6). Quercetin, at a concentration of 1 *μ*M, did not change total de novo synthesis in canine blood. However, treatment of canine blood with 10 *μ*M quercetin caused a significant increase in de novo protein synthesis compared to the vehicle control. Because HRI is specifically expressed in erythroid cells and ~95% of de novo protein synthesis in late erythroblasts and reticulocytes is globin protein [12], the newly synthesized [^35^S]-methionine labeled protein measured is most likely due to de novo hemoglobin synthesis in erythroid cells. Therefore, we conclude that inhibition of HRI by the HRI kinase inhibitor is able to increase hemoglobin synthesis in canine reticulocytes.

## 4. Discussion

Hemoglobin synthesis in erythroid cells is tightly regulated by heme, because heme binds to HRI and modulates its kinase activity. It has been reported that the enzymatically functional form of HRI is dimeric [7] and remains soluble devoid of aggregation. Maintaining the dimeric form of HRI in order to prevent its aggregation makes HRI a difficult protein to express in large quantities in vitro. We found that HRI is largely misfolded when expressed by in vitro TNT, however, the expression of soluble HRI protein can be improved by supplementing the reaction with heme (Figure 2). In the HRI protein expression conditions used in this study, heme primarily plays a critical role by inactivating newly synthesized HRI by binding to the kinase insert domain. Consequently, heme addition increased protein production. In addition, heme addition appears to improve the yield of soluble protein by preventing newly translated HRI protein from aggregation. It is conceivable that HRI incorporates a heme moiety into the N-terminus of the protein cotranslationally to allow for the proper folding and assembly of the protein. To improve the protein yield of the human, mouse, rat, and canine HRI proteins, we supplemented insect cell culture medium with hemin during HRI protein expression (Figure 3). 

A comparison of the mammalian HRI nucleotide and amino acid sequences reveals a high degree of conservation in the residues involved in ATP-binding (Figures 1(c), 1(d), and 1(e)). Specifically, the comparison shows there is only one amino acid residue difference between the human and canine proteins and only two residues between the mouse and canine proteins. Additionally, the residues involved in heme-binding, either in the N-terminal regulatory domain or in the kinase insert domain, are completely conserved between the mammalian proteins, implying that the development and homeostatic regulation of circulatory systems among mammals is subject to the same evolutionary pressure (Figure 1(d)). As expected from the high degree of sequence homology, we have found that the mammalian HRI proteins show similar ATP affinity with an apparent *K*_*m*_ of ~1-2 *μ*M (data not shown). The high degree of homology in the catalytic domain is also reflected by the similar potency determined between the mammalian proteins in the ability of quercetin to inhibit HRI kinase activity (Figure 5). 

Normal adult hemoglobin consists of two *α*- and two *β*-globin subunits and its proper assembly is essential for oxygen transport [5]. The majority (>90%) of actively translating mRNA in reticulocytes is globin mRNA, and 95% of protein made in reticulocytes is globin protein [12]. Translation of *α*- and *β*-globin is tightly controlled by eIF2 and downregulated by HRI. By enhancing translation of globin by an HRI inhibitor in reticulocytes, the hemoglobin content of red blood cells is expected to increase causing an increase in the body's oxygen-carrying capacity. In this study, we show that inhibition of HRI by quercetin is able to increase de novo protein translation in canine reticulocytes (Figure 6) and also in mouse and rat reticulocytes (data not shown). Since HRI is predominantly expressed in erythroid cells [5] and since mature red blood cells do not have the ability to synthesize protein, we used the [^35^S]-methionine incorporation in canine blood as an indirect measure of hemoglobin synthesis in erythroid cells. Our finding that HRI inhibition increases hemoglobin synthesis leads us to propose that antagonism of HRI may be of potential therapeutic importance for the treatment of iron-deficient blood disorders. 

This study also shows that the canine HRI is conserved not only in the primary sequence of the gene but also functionally in its role on the regulation of protein synthesis. Together with the advantage of an ample supply of blood and similar physiology between canine and human, canine appears to be a good model species for further elucidation of the role of HRI protein in its contribution to iron-deficient anemias and perhaps other hematologic diseases such as *β*-thalassemia.

## Abbreviations

eIF: Eukaryotic initiation factor
HRI: Heme-regulated inhibitor
ATP: Adenosine tri-phosphate
TNT: Transcription and translation
Quercetin: 3,3′,4′,5,7-pentahydroxyflavone
TCA: Trichloroacetic acid
bp: Base pair.
